# Oral Immunotherapy in Food Allergy: A Critical Pediatric Perspective

**DOI:** 10.3389/fped.2022.842196

**Published:** 2022-02-22

**Authors:** Aysegul Akarsu, Giulia Brindisi, Alessandro Fiocchi, Anna Maria Zicari, Stefania Arasi

**Affiliations:** ^1^Division of Allergy and Asthma, Department of Pediatrics, Hacettepe University Faculty of Medicine, Ankara, Turkey; ^2^Department of Maternal and Child Health and Urological Sciences, Sapienza University of Rome, Rome, Italy; ^3^Translational Research in Pediatric Specialities Area, Division of Allergy, Bambino Gesù Children's Hospital (IRCCS), Rome, Italy

**Keywords:** children, cow's milk allergy, desensitization, food allergy, egg allergy, oral immunotherapy, peanut allergy, sustained responsiveness

## Abstract

There is evidence that in children with persistent IgE-mediated food allergy (FA) to cow's milk, hen's egg, and peanut, oral allergen-specific immunotherapy (OIT) may increase the reaction threshold to the culprit food allergen(s). OIT may protect patients from the occurrence of severe reactions in case of accidental ingestion of the culprit food during treatment. Notwithstanding, many gaps are still unsolved, including safety issues, identification of predictive biomarkers, and post-desensitization efficacy. In this perspective, the use of omalizumab (Anti-IgE monoclonal antibody) has been proposed as an adjunctive treatment to OIT in order to reduce the risk of allergic reactions related to OIT. This review aims to summarize the current evidence and unmet needs on OIT in children with FA to enhance the development of longitudinal, prospective, and well-designed studies able to fill the current gaps soon.

## Introduction

Immunoglobulin E (IgE) mediated food allergies (FA) represent an adverse and potentially life-threatening condition caused by the exposure to a specific food allergen through an immediate IgE-mediated immunological mechanism (type 1 of Gell and Coombs) (1). Based on the underlying patho-mechanism, and specifically on the involvement of a hypersensitivity reaction, FA are generally classified into immunoglobulin (Ig)E-mediated FA, non-IgE mediated, and mixed ones (2). In the last few decades, reports show that FA prevalence has been increasing in industrialized countries (2–4). Globally the estimated incidence of FA ranges from 0.45 to 10% in infants and preschool-aged children, from 1 to 5% in school age, and about 4.5% in adult age (5–7). Although the majority of FA reactions are mild-moderate, they sometimes are severe and even fatal or near-fatal (8–12). Referring to retrospective case series, the fatality rate is estimated between 0.65 and 2% (13, 14).

The most allergenic foods are milk, egg, peanut, tree nut, wheat, soy, fish, and shellfish with a prevalence related to the age and local dietary habits (2, 4, 15–18). The natural history of FA is variable: the majority of children with allergies to egg, milk or soy allergy overcome their FAs, vice versa, FAs to peanuts, tree nuts, and seafood are more difficult to be resolved (18). Overall, for egg, tolerance is reached by 3 years of age in the 50% of cases and by school-age in 80%; for milk it is achieved by 5 years of age in about 50% of cases (19). Less than 10–20% of allergies to peanut or tree nuts achieves spontaneous clinical tolerance (20).

The negative impact of FAs on pediatric patients and their families' lives may be significant due to several reasons including: difficulties in practicing a strict allergen avoidance; possible nutritional impairment; fear of accidental exposure; feeling of being different from one's peers; absences from school and from work, respectively, for patients and their parents which generate a major detriment to a country's economy (21–27).

According to international guidelines, the current standard of care in the management of FA is the strict elimination diet and the use of adrenaline as rescue medication in case of severe allergic reactions, such as anaphylaxis (28). Alternative treatment strategies to the avoidance diet have been investigated; the most promising therapeutic strategies are currently oral immunotherapy (OIT) and biologicals (such as omalizumab) (29–31). Other routes of administrations other than the oral [such as epicutaneous (EPIT, epicutaneous immunotherapy) and sublingual ones (SLIT, sublingual immunotherapy)] have been investigated. Although their safety profile appears to be good, efficacy seems to be lower in both in magnitude and in number needed to treat than for OIT. Furthermore, there are no products for EPIT currently available on the market and none has been approved by a regulatory authority. This review aims to critically provide an overview on the current evidence and unmet needs related on OIT in children suffering from FA.

## General Concepts on Allergen Immunotherapy

**Figure 1:**
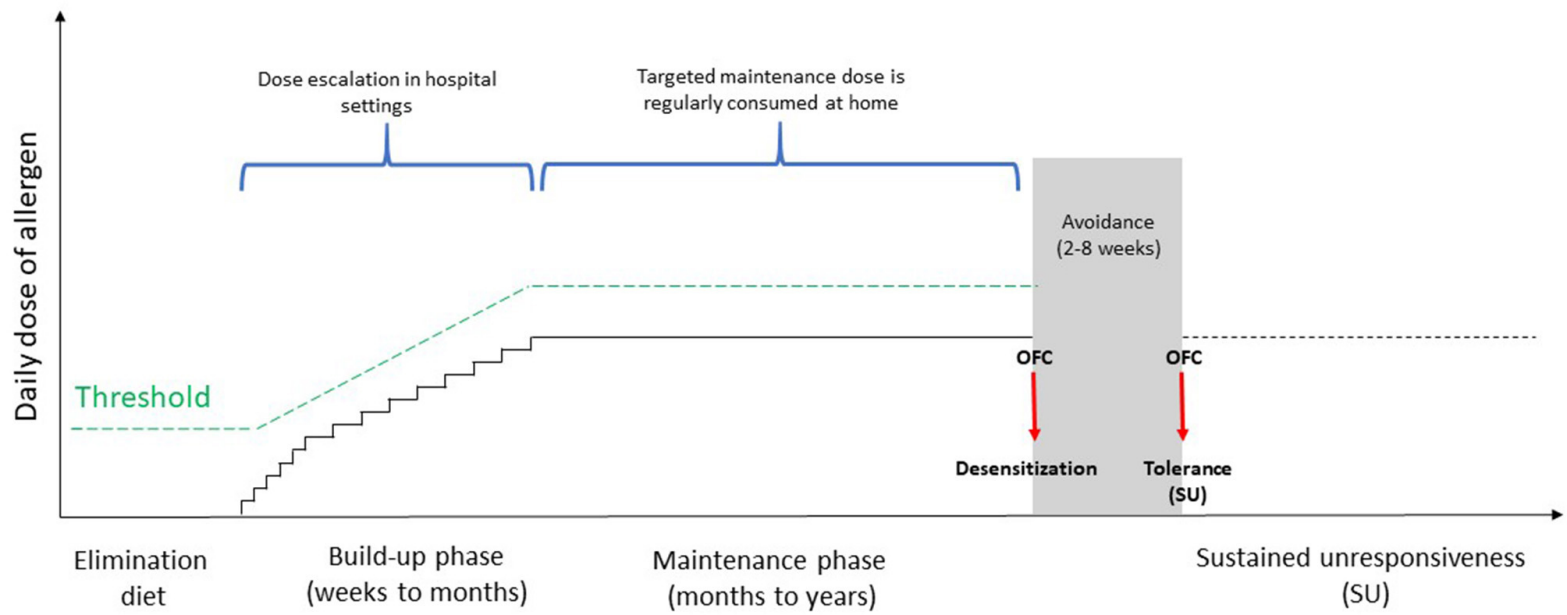
Classic protocol of oral immunotherapy. OFC, oral food challenge.

OIT consists of a titrated oral administration of the culprit food at regular intervals to induce tolerance [i.e., the possibility to take unlimited amounts of the culprit food without presenting reactions even after its intake is stopped indefinitely], starting with a build-up phase where increasing quantities of the food are administered in hospital. Usually, during the build-up phase the maximum tolerated dose is assumed daily at home in the interval during dose increases, usually on weekly or every other week basis. The build-up phase is followed by a maintenance phase with regular, daily intake of a maximum tolerated amount of food (32) (Figure 1). The protocols are heterogeneous and differ in relation to the type of food used (e.g., fresh or baked), the number of doses administered, the amount of allergenic protein per dose, the framework between the single doses and the maintenance one. OIT, as stated by the European Academy of Allergy and Clinical Immunology (EAACI), represents the only potentially curative treatment for FA so far, capable of modulating the immune system and modifying the natural history of disease (33). The primary aim of OIT is the increase of reactivity threshold in order to prevent patients from life-threatening events due to accidental ingestion of the culprit food (34, 35). The clinical effectiveness of OIT is commonly evaluated in terms of “desensitization” [i.e., an increase in the threshold of reactivity toward a specific food, allowing the patient to consume the culprit food without adverse reactions while continuing OIT (35–38)] and sustained unresponsiveness (SU) [i.e., the possibility to assume any amount of the incriminated food, even after a long period of its avoidance (34, 36, 39, 40)] (Figure 1). Factors associated with a greater chance of achieving SU include a longer duration of maintenance phase and younger age for commencing OIT (39). To date, there are no guidelines stating the perfect time when OIT should be discontinued prior to demonstrate SU but commonly it is a framework of 4–8 weeks. In addition, the length of time that SU must persist to confirm the achievement of tolerance, remains unknown, and it seems that this state may not be long-lasting after OIT (41).

## Mechanisms of Allergen Immunotherapy

**Figure 2:**
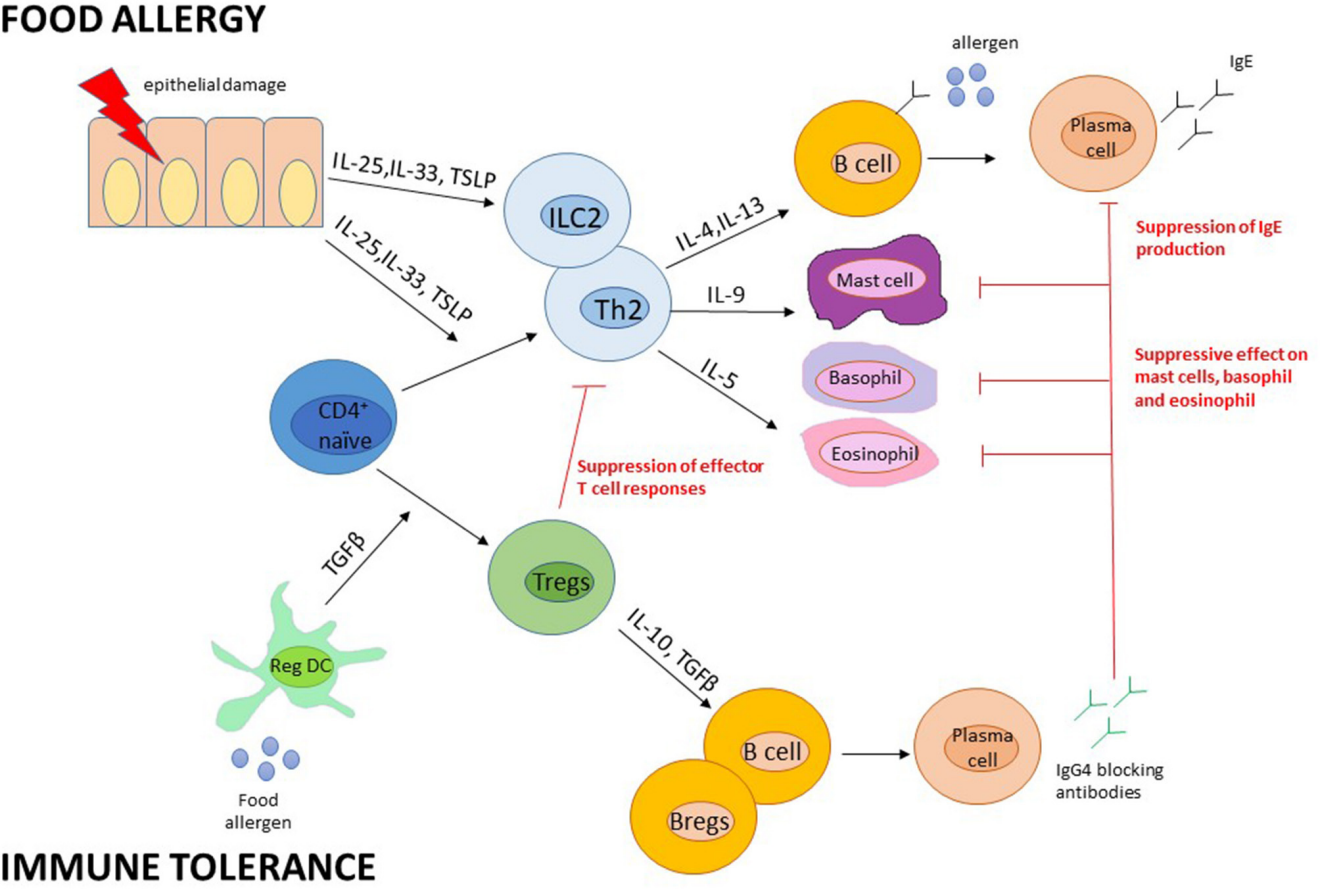
Presumed immune mechanisms in food allergy and immune tolerance. Adapted from Pajno et al. (43). B regs, B regulatory cells; DC, dendritic cells; Ig, Immunoglobulin; IL, interleukin; ILC, Innate Lymphoid cells; T regs, T regulatory cells; TSLP, thymic stromal lymphopoietin.

The mechanisms of action are not fully understood. It has been shown a reduction of specific IgE levels (after an initial raise) followed by an increase in specific immunoglobulin G4 (IgG4), reaching a balance in the achievement of tolerance. IgG4 compete with sIgE for allergen binding, suppressing the reactivity of mast cell and basophils, which are deprived of their preformed mediators through continuous degranulation (42, 43) (Figure 2).

During OIT, thanks to the constant low dose allergen exposure, it is observed a reduced basophils activation with a global state of their hyporesponsiveness or anergy, not related to IgE levels. In fact, IgE generally increase after the onset of OIT, then remain elevated for months until they fall to baseline or lower levels. However, this OIT-induced basophils suppression is not definitive but seems to be necessary to maintain remission after OIT discontinuation (39).

Poor data are available on the role of mast cells in OIT, which has a long half-life that lasts from months to years. Recent studies on murine models, suggest that early degranulation of mast cells may have a pivotal role in desensitization and, similar to basophils, it provides a defense against allergens exposure (44, 45).

So it is reasonable to think that the suppression of mast cells and basophils activity due to continuous exposure to low-dose allergen is the basis of desensitization during OIT, and that the depletion of this suppression is related to the gradual reappearance of clinical reactivity in some patients after OIT suspension (39).

In OIT, the spotlight has been on T regulatory (Treg) cells with the conversion of allergen-specific T cells to anergic T cells, involving the epigenetic regulation of the FOXP3 (forkhead box P3) gene in allergen-specific Treg cells (46).

## Potential Biomarkers

There is still a long way to find reliable biomarkers that can predict good responders to OIT and personalize the protocol schedule, including the duration of SU.

Some studies have shown that low skin prick tests' (SPTs) wheals diameters and serum sIgE at baseline and at the end of maintenance phase besides a reduced basophil reactivity may predict SU achievement; while IgG4 are not predictive of SU (39).

The regulatory markers, in particular FOXP3+ and latency associated peptide (LAP+) Tregs, seem to play a key-role in inducing long-term tolerance in patients successfully treated with OIT (47).

## Contraindications to OIT

The major OIT contraindications include: non-IgE mediated allergy; uncontrolled asthma; treatments contraindicating adrenaline, low family compliance (33, 48). Further criticisms in OIT come from the lack of standardized protocols, the spontaneous development of tolerance especially for cow's milk and egg, the need for patients' compliance and the possibilities of side effects as well as the requirement of the availability of trained health care professionals, appropriate clinical facilities to provide OIT and deal with adverse effects (35, 49, 50). Decision aids might help individuals (and their parents) make decisions consistent with their values and preferences.

## Cow'S Milk OIT

EAACI guidelines suggest to start OIT in children when they are about 4–5 years old because at this age 50–90% of them have already outgrown their allergy (33). Nevertheless, an early intervention, especially in children affected by a severe cow's milk allergy (CMA), is considered to be more effective (32).

Overall there is moderate-to-strong evidence on its benefit in terms of desensitization although with a higher risk of AEs, mainly mild to moderate (51–60) (Supplementary Table 1A). However, the risk of serious side effects should not be overlooked and a case of death due to this procedure has recently been reported in a non-scientific ambit.^1^ Results from an updated SR by the Diagnosis and Rationale for Action Against Cow's Milk Allergy (DRACMA) project are submitted.

Among the several studies reported, Longo et al. showed that rush a OIT protocol (i.e., an initial rush up-dosing phase with multiple increasing doses for 10 days in a hospital setting, followed by a slow increasing phase at home) in 97 children affected by severe CMA was effective and safe with data similar to a slower procedure (61). A weekly-up dosing OIT in 33 children with severe CMA over 4 months was reported to be an alternative method to achieve desensitization, being less time consuming than every other week up-dosing regimen and overall safe if performed in a well-equipped hospital setting (62). Factors that might predict adverse reactions during OIT are milk specific IgE levels, wheal size at SPTs, concomitant asthma / eczema or history of anaphylaxis (63–66).

The knowledge on SU is less robust. Factors that have been speculated to be linked to SU include the duration of maintenance phase (67). Biomarkers useful for the prediction of SU in CMA are the initial lower milk specific IgE levels, a small wheal diameter of the SPTs and a low basophil activity toward allergens (39, 68, 69). OIT in CMA induces also a reduction in the avidity of IgE and an increase of IgG4 binding to milk protein epitopes, resulting in a greater likelihood of obtaining SU (68, 70, 71). Nevertheless, the lack of acquisition regarding SU achievement in all treated subjects with CMA may underlie significant differences in individual immune systems, and further studies are desirable in the future to better understand these mechanisms.

## Egg OIT

From the first egg-OIT reported in 1908 (72), several studies have shown its effectiveness and safety (73–89) (Supplementary Table 1B). The form of the egg ingested during OIT as well as the definition and rate of desensitization, the OIT protocol, the primary outcome were different between studies (36, 75, 79, 80, 82, 86) (Supplementary Table 1B). In the first double blind placebo-controlled food challenge (DBPCFC) egg-OIT study published (75), fifty-five egg-allergic children received OIT, consumed 2 g egg-white powder in maintenance phase and 55 and 75% of the OIT group passed oral food challenge (OFC) at 10 and 22 months of treatment, respectively. None of the patients in placebo group passed OFC. At 22 months, OIT was discontinued and children were instructed to avoid all egg consumption for 4–6 weeks. At 24 months, 28% of OIT group passed OFC, reaching SU. All children who had SU were consumed egg without any allergic reaction at 30 and 36 months. Long-term results of the study were reported by Jones et al. demonstrating that 50% of the OIT group had SU by 4 year (84). Kim et al. investigated the safety and efficacy of egg-OIT compared with baked egg (BE) consumption in children (aged 3–16 years) who were BE-tolerant but unbaked egg reactive (88). They concluded that OIT was more effective to achieve SU than ingesting BE alone.

## Peanut OIT

The first open-label studies about peanut-OIT were published in 2009 (90, 91) followed by several studies in last decades (92–109) (Supplementary Table 1C). In STOP II study, after 6 months of OIT with 800 mg peanut protein (PP) maintenance dose, 24/39 children (62%) passed DBPCFC of 1,400 mg PP. No patients (*n* = 46) in control group (avoidance) passed DBPCFC. In the crossover phase of the study, 45 children from control group started OIT and 54% passed OFC of 1,400 mg at the end of the therapy (97). No serious adverse events were reported during treatment (97). In 2017 Vickery et al. compared the efficiency and safety of low-dose (300 mg PP, *n* = 20) and high dose (3,000 mg PP, *n* = 17) peanut-OIT in pre-school age children (100). They reported that the desensitization (85 vs. 75%) and SU rate (85 vs. 71%) did not differ significantly between low-dose and high-dose groups. Moreover, high-dose group experienced higher rate of moderate-to-severe adverse event than low-dose group. In 2018, PALISADE study investigated a standardized maintenance dose (phase 2 trial of AR101, 300 mg peanut PP) peanut OIT (103). They reported that 62% of OIT group passed OFC of 1,043 mg while no children in placebo group could pass it. After positive results of phase 2 trial, the outcome of phase 3 trial of AR101 was reported by Vickery et al. (105). In this multicenter study, 496 children underwent peanut-OIT using AR101 (standardized 300 mg PP). After 12 months maintenance period, 67% of OIT group passed OFC of 600 mg PP, whereas the rate was 4% in placebo group. Most patients (60%) had mild to moderate adverse events and 4.3% of the subjects reported severe adverse events. Besides, authors reported effectivity and safety of alternative dosing regimens from 2-year follow-on study of PALISADE participants (109). They observed the highest desensitization rate in the group who had longest daily dosing duration (300 mg/daily during 24–56 weeks) (Supplementary Table 1C). In addition, adverse events (AEs) rates were higher in non-daily dosing cohorts than daily cohorts and most children had mild/moderate AEs. Then, in January 2020, Palforzia (AR101), which is a standardized peanut OIT formulation approved by US Food and Drug Administration (FDA), became the first drug approved for OIT in FA treatment (110).

## OIT for Other Foods

Few reports describe OIT to other foods, including tree nut, sesame, cashew and wheat (111–122) (Supplementary Table 1D). In the first reported walnut-OIT study (111), 73 patients were randomized in OIT (*n* = 55) group and control group (*n* = 18). At the end of the study, 89% of OIT group passed OFC of 4,000 mg walnut protein and all children co-allergic to pecan achieved desensitization to pecan. In addition, 60 and 93% of the patients co-allergic to hazelnut/cashew and hazelnut alone achieved desensitization, respectively. During the study, most of the patients (85%) experienced AEs mostly mild-moderate and intramuscular adrenaline was administered to 9 children.

## Adjunctive Treatments to OIT

Omalizumab has been used as an adjuvant during OIT protocol to reduce risk of allergic reactions. In milk and egg OIT, Omalizumab reduced the number and severity of reactions during dose-escalation phase and allowed a rapid build-up phase (59, 123–125). However, no additional effect was found to achieve desensitization with omalizumab (59, 124). Similar effects have been described for peanut OIT, (95, 99, 126, 127) even in high-risk patients (95). In addition, the effectiveness of Omalizumab in multiple food allergens-OIT were comparable to the outcomes of a single food OIT (121, 128). However, the effects on efficacy and safety of dose adjustment, according to body weight and total IgE levels, or in fixed doses are uncertain. Hence, the duration, dosage, and effectiveness of Omalizumab treatment in OIT remains to be clarified. In particular, one of the main gaps in knowledge lies on the effectiveness after the discontinuation of omalizumab.

## Discussion and Future Perspectives

FA is a major health problem with growing prevalence (129). The main treatment option of FA is dietary restriction, using rescue medications in case of severe allergic reactions (130). Moreover, novel therapies for FA treatment including microbiome, biologic agents, oral/sublingual/subcutaneous/epicutaneous immunotherapy (IT) were reported in the last decades (29, 50, 131, 132). While results of recent OIT studies are encouraging, the major issue of OIT is the heterogeneity of study protocols including the duration of maintenance doses, primary end points, definition of desensitization, OFC protocols to evaluate desensitization, SU and safety profiles (35). The natural raw form of food was usually used in OIT (51, 62, 71, 78). On the other hand, some studies revealed with processed food allergens, such as hydrolyzed, pasteurized, dry powdered, heated, undercooked, etc. (36, 75, 79, 80, 82, 86). In peanut OIT, the first standardized product protein (Palforzia, AR101, containing 300 mg PP) has been recently approved by FDA (110). Molecular IT for tree nut and peanut allergies might be another treatment option in the future, along with developments in the diagnosis of molecular allergy (133, 134).

Although, most of the reactions are mild to moderate and occur mostly during the build-up phase performed in clinical settings, allergic reactions may appear also during maintenance phase at home (33). A rare but important side effect of OIT is eosinophilic esophagitis (EoE) with a frequency rated of 0.3%, instead 8.3% of patients experiences gastrointestinal symptoms during OIT (135, 136). Further, two meta-analysis reported that OIT increases anaphylaxis risk and frequency of adrenaline use (35, 137). Of importance, two cases of lethal reaction to the intentional introduction of a food are reported^1^ (138). Although this can be considered an extremely rare eventuality, the clinician must inform the family of this possibility at the beginning of each treatment with OIT.

Several efforts are under investigation in order to improve the safety profile, including the slow introduction [i.e., slow progression schedule starting with baked food (e.g., milk/egg) and then less and less heated food over time (with a presumed lower risk of side-effects] (139) and low dose introduction (140). The main goal of OIT is an increase in the allergen reactivity threshold to achieve lower risk of severe allergic reactions after accidental ingestion (33). Accordingly, it is expected that patients have less fear of anaphylaxis after allergen exposure, less restriction in social life, and a consequent increase in their food allergy-related quality of life (FA-QoL) (27, 86, 97, 106, 141–147). However, the current data are not sufficient to make definitive conclusions about the impact of OIT on QoL and more studies are needed.

Another issue that should be highlighted is the cost-effectiveness of the treatment, recently investigated only for peanut OIT and EPIT (148–150). These studies reported that OIT and/or EPIT may be cost-effective in certain conditions: lower therapy cost, achieving SU, improvement in health state utility, and reduction of anaphylaxis risk (149, 150). However, further well-designed studies are needed to better explain health state improvement in OIT (150).

In conclusion, OIT is a promising treatment in FA and it will be important to define standardized protocols, considering also the possible use of Omalizumab as an adjuvant therapy. Understanding of the mechanism associated with remission or SU is fundamental, thus reaching the goal of therapy.

## Author Contributions

SA designed the manuscript. AA and GB wrote the first draft. SA provided background, coordinated, and supervised the work. All authors critically revised the work and contributed to the article, approving the submitted version.

## Conflict of Interest

The authors declare that the research was conducted in the absence of any commercial or financial relationships that could be construed as a potential conflict of interest.

## Publisher's Note

All claims expressed in this article are solely those of the authors and do not necessarily represent those of their affiliated organizations, or those of the publisher, the editors and the reviewers. Any product that may be evaluated in this article, or claim that may be made by its manufacturer, is not guaranteed or endorsed by the publisher.

## Footnotes

^1^Available online at: https://www.allergicliving.com/2021/12/20/girl-with-milk-allergy-dies-of-severe-reaction-related-to-desensitization/?s=09 (accessed December 22, 2021).

## Abbreviations

AE: Adverse event
CMA: Cow's milk allergy
DBPCFC: Double blind placebo-controlled food challenge
DRACMA: Diagnosis and Rationale for Action Against Cow's Milk Allergy
EAACI: European Academy of Allergy and Clinical Immunology
EoE: Eosinophilic esophagitis
EPIT: Epicutaneous immunotherapy
FA: Food allergy
FA-QoL: Food-allergy-related quality of life
FDA: Food and Drug Administration (FDA)
FOXP3: Forkhead Box P3
IgE: Immunoglobulin E
IgG4: Immunoglobulin G4
IT: immunotherapy
LAP: Latency associated peptide
OFC: Oral food challenge
OIT: Oral immunotherapy
PP: Peanut protein
SPTs: Skin prick tests
SLIT: Sublingual immunotherapy
SU: Sustained unresponsiveness
Treg cells: T regulatory cells.
