# Supplementary material for: Oral Immunotherapy in Food Allergy: A Critical Pediatric Perspective
Source: Front Pediatr. 2022 Feb 22;10:842196. doi: 10.3389/fped.2022.842196 (PMC8901728; doi:10.3389/fped.2022.842196)
Supplement: Supplementary file 1 [file Table_1.DOCX]

| 1. **Cow’s milk OIT studies** | | | | | | | | | | | | | | | | |
| --- | --- | --- | --- | --- | --- | --- | --- | --- | --- | --- | --- | --- | --- | --- | --- | --- |
| **Study** | **Allergen** | | **Study Design** | **Number of participants** | | | **Patients’ age, median/mean**  **(range)** | | **Maintenance dose** | | **Duration** | | **Results (Desensitization / Intention to treat)** | | **Sustained unresponsiveness (SU)** | |
| Longo et al, 2008 (61) | Cow’s  milk | | DBPCFC | AG: 30 (OIT)  CG:30 (avoidance) | | | 5-17 years | | 150 ml | | 12 months | | AG: 11/30 (36%) achieved oral desensitization  16/30 (54%) achieved partial desensitization  3/30 (10%) failed for the appearence of severe reactions  CG: 100%  failed the achievement of tolerance | | NA | |
| Skripak et al, 2008 (55) | Cow’s milk | | DBPCFC | AG: 12  CG:7 | | | 6-17 years | | 15 ml (5140 mg) | | 23 week | | AG: 100% reached a full tolerance at 5140 mg  CG:100% reached a tolerance of less than 40 mg | | NA | |
| Caminiti et al, 2009 (62) | Cow’s milk | | Randomized, placebo controlled | AG: 7 (OIT in open fashion)  CG:6 (double blind placebo controlled) | | | 5-10 years | | 200 ml | | 4 months | | AG: 5/7 (71.4%) achieved full desensitization (200 ml)  1/7 (14.3%) achieved partial desensitization (64ml)  1/7 (14.3%) failed  CG: 3/6 (50%) showed a positive food challenge | | NA | |
| Pajno et al, 2010 (53) | Cow’s milk | | DBPCFC | AG: 15  CG: 15 (DBPCFC) | | | 9 years  (4-12 years) | | 200 ml | | 18 weeks | | AG: 10/15 (66.7%) achieved full tolerance (200 ml)  1/15 (6.7%) achieved partial tolerance  2/15 (13.3%)failed for the appearence of severe reac tions  2/15 (13.3%) drop out for personal reasons  CG: 1/15 (6.7%) drop out for personal reasons  14/15(93.3%) failed the achievement of tolerance | | NA | |
| Martorell et al., 2011 (59) | Cow’s  milk | | Randomized controlled | AG: 30  CG: 30 (avoidance) | | | 24-36 months | | 200 ml | | 12 months | | AG: 27/30 (90%) achieved oral desensitization (200 ml)  CG: 7/30 (23% achieved oral desensitization | | 12 months  in the AG | |
| Lee et al, 2013 (71) | Cow’s  milk | | DBPCFC | AG: 16  CG:15 (avoidance) | | | 7-12 months | | 200 ml | | 6 months | | AG: 14/16 (87.5%) achieved oral desensitization (200 ml)  2/16 (12.5%) failed for the appearence of severe reactions  CG: 12/15 (80%) failed the achievement of tolerance  3/15 (20%) excluded for personal reasons | | NA | |
| Salmivesi et al, 2013 (54) | Cow’s milk | | DBPCFC | AG: 18  CG: 10 | | | 6-14 years | | 200 ml | | 23 weeks | | AG: 16/18 (89%) successfully completed the protocol  2/18 (11%) failed the achievement of tolerance for the appearance of symptoms  CG:8/10 (80%) successfully completed the protocol  1/10 (10%) anaphylaxis  1/10 (10%) drop out for personal reasons | | > 3 years | |
| Takahashi et al, 2017 (118) | Cow’s milk | | Randomized, controlled | AG: 10 (omalizumab+OIT)  CG: 6 | | | 6-14 years | | 200 ml | | 6 months | | AG: 100% achieved desensitization  CG: 100% did not develop desensitization | | NA | |
| 1. **Hen’s egg OIT studies** | | | | | | | | | | | | | | | | |
| Buchanan et al, 2007 (73) | Egg | | Open label | 7 | | | 44.7 months (14-84 months) | | 300 mg EP | | 24 mo | | 4 children (57%) passed the 8 gr-OFC | | Avoid time: 3 mo  2 children (29%) passed 8 gr-OFC | |
| Vickery et al, 2010 (74) | Egg | | Open label | 8 | | | 5 years  (3-13 years) | | 300 - 3600 mg EP | | 18-50 mo | | 2 children withdrew | | Avoid time: 1 mo  6 children (75%) passed OFC | |
| Burks et al, 2012 (75) | Egg | | Randomized, placebo controlled | AG: 40  CG: 15 (placebo) | | | 7 years  (5-11 years) | | 2 gr egg white powder (equivalent of 1/3 of an egg) | | 22 mo | | AG:  After 10 months:  22 children (55%) passed OFC (5 gr egg-white powder)  After 22 months:  30 children (75%) passed OFC (10 gr egg-white powder).  CG: None of the children passed OFC | | Avoid time: 4-6 w  1 child withdrew  11 children (28%) passed OFC. (10 gr egg white powder plus 1 whole cooked egg). | |
| Dello Iacono et al, 2013 (76) | Egg | | Randomized, controlled | AG: 10  CG: 10 (avoidance) | | | 7 years  (5-11 years) | | NA  Increasing doses (up to 40 ml) | | 6 mo | | AG: 9/10 children (90%) achieved partial tolerance (10-40 ml)  None of the children achieved maximum tolerance (≥40 ml)  CG: 1/10 child could tolerate more than 1 mL (1.8 mL) | | NA | |
| Fuentes- Aparicio et al, 2013 (77) | Egg | | Randomized, controlled | AG: 40  CG: 32 (avoidance) | | | 8.7 years  (4-15 years) | | 2 eggs/week | | 2.5 mo | | AG: 37 children (92.5%) achieved desensitization.  20 children (50%) passed OFC with raw egg white.  CG: 7 children (21.8%) reached spontaneous tolerance. | | NA | |
| Meglio et al, 2013 (78) | Egg | | Randomized, controlled | AG: 10  CG: 10 (avoidance) | | | 8 years  (4-14 years) | | 25 ml raw egg (13.6 g EP) | | 7 mo | | AG: 8 children (80%) achieved 25 ml raw egg  1 child (10%) tolerated 2ml raw egg but can eat 15 g cooked egg.  CG: 2 children (50%) reached spontaneous tolerance after 6 months. | | NA | |
| Caminiti et al, 2015 (79) | Egg | | Randomized, placebo controlled | AG: 17  CG: 14 (Placebo) | | | 6 years  (4-11 years) | | 4 g dehydrated egg white | | 4 mo  plus  6 mo egg-containing diet (2-3 eggs per week) | | AG: 16 children (94%) achieved desensitization  CG: 1 child (7.1%) reached spontaneous tolerance after 13 months. | | Avoid time: 3 mo  5 children (29.4%) passed OFC (3.7 g egg white) | |
| Escudero et al, 2015 (80) | Egg | | Randomized, controlled | AG: 30  CG: 31 (avoidance) | | | 8 years  (5-17 years) | | One undercooked egg | | 3 mo | | AG: 28 children (93.3%) achieved desensitization  CG: 1 child (3%) reached spontaneous tolerance after 4 months. | | Avoid time: 1 mo  11 children (37%) passed 2.8 g EP-OFC | |
| Yanagida et al, 2016 (81) | Egg | | Randomized, controlled | AG:21  CG:12 (avoidance) | | | 9 years  (6-19 years) | | 194 mg heated whole egg (1/32 of a whole egg) | | 12 mo | | AG: 16 children (76%) achieved desensitization  CG: None of the children passed 194 mg heated egg-OFC. | | Avoid time: 2w  15 children (71.4%) passed 194 mg heated egg.  7 children (33.3%) passed ½ of whole egg-OFC. | |
| Giavi et al, 2016 (82) | Egg | | Randomized, placebo controlled | AG: 15  CG: 14 (placebo) | | | 2 years  (1-5 years) | | 9 g hydrolysed egg | | 6 mo | | AG: 4 children (26.7%) passed 43.2 g boiled egg-OFC.  4 children withdrew  CG: 3 children (21%) passed 43.2 g boiled egg-OFC. | | NA | |
| Akashi et al, 2017 (83) | Egg | | Randomized, controlled | AG: 18  CG: 18 (avoidance) | | | 6 years  (4-11 years) | | 4 g dry powdered egg | | 6.5 mo | | AG:  8 children (44.4%) reached 4 g, 5 reached 3 g, 1 reached 1.4 g.  4 withdrew  CG:  16 children failed 4g-OFC.  2 withdrew | | NA | |
| Jones et al, 2016 (84) | Egg | | Randomized,  placebo controlled | AG: 40  CG: 15 (placebo) | | | NA  (5-18 years) | | 2 gr egg white powder | | 48 mo | | AG:  2^nd^ year: 30 children (75%) achieved desensitization.  3^rd^ year: 31 children (77.5%) achieved desensitization.  4^th^ year: 31 children (77.5%) achieved desensitization.  CG: None of the children passed 10g-OFC after 44 weeks.  2 children withdrew | | Avoid time: 4-6 w  AG:  2^nd^ year: 11 children (27.5%) passed 10 g & whole egg-OFC.  3^rd^ year: 18 children (45%) passed 10 g & whole egg-OFC.  4^th^ year: 20 children (50%) passed 10 g & whole egg-OFC. | |
| Perez-Rangel et al, 2017 (85) | Egg | | Randomized, controlled, cross-over | AG: 19  CG: 14 (avoidance) | | | 10 years  (5-18 years) | | One undercooked egg every 48 hours | | 5 mo | | 30 children (86%) achieved desensitization.  3 children withdrew.  CG: None of the children passed 2800 mg- OFC | | NA | |
| Itoh- Nagato et al, 2018 (86) | Egg | | Randomized, controlled | Early-start group: 23  Late-start group: 22 | | | 7 years  (5-15 years) | | 60 g of cooked egg and 1 g egg white powder | |  | | Early- start group: 20 children (87%) achieved an increase in the threshold dose of egg white powder  Late-start group: 5 children (22.7%) achieved an increase in the threshold dose of egg white powder | | NA | |
| Martin-Munoz et al, 2019 (87)  (SEICAP I study) | Egg | | Randomized, controlled | AG: 76  CG: 25 (avoidance) | | | 6-9 years | | 30 mL pasteurized egg white (3.3 g protein) | | 12 mo | | AG: 64 children (84.2%) achieved desensitization  CG: 4 children (16%) reached spontaneous tolerance.  12 children underwent OIT and 8 out of them achieved desensitization.  Total desensitization rate: 72/88 (82%) | | Avoidance: *ad libitium*  6 months after: 47 children (69.3%) passed 3.3g protein-OFC.  12 months later: 46 children (52.3%) passed 3.3g protein-OFC. | |
| Kim et al, 2020 (88) | Egg | | Randomized, controlled | AG: Baked egg tolerant (BE-T): 23  Baked egg reactive (BE-R): 39  CG: 27 (baked egg) | | | 7.3 years  (3-16 years) | | OIT: 2.5 g dried standard egg white powder (2 g egg white protein)  CG: a muffin (2 g egg white protein) | | 24 mo | | 1^st^ year:  AG:  BE-T: 20 children (87%) achieved desensitization.  3 children withdrew  BE-R: 34 children (87%) achieved desensitization.  5 children withdrew  CG: 23 children (85.2%) achieved desensitization.  4 children withdrew  2^nd^ year:  AG:  BE-T: 18 children (78.3%) passed OFC. (7444 mg egg white protein)  BE-R: 21 children (54%) passed OFC. (7444 mg egg white protein)  CG: 4 children (15%) passed OFC. (7444 mg egg white protein) | | Avoid time: 8-10 w  AG:  BE-T: 10 children (44%) achieved SU. (7444 mg egg white protein)  BE-R: 7 children (18%) achieved SU. (7444 mg egg white protein)  CG: 3 children (11.1%) achieved SU. (7444 mg egg white protein) | |
| Palosuo et al, 2021 (89) | Egg | | Randomized, controlled, cross-over | AG: 33  CG: 23  (avoidance) | | | 11 years  (6-17 years) | | 1 g egg white protein | | 8 mo | | CG: 1 child  reached spontaneous tolerance after 6 months  5 children withdrew  50 children underwent OIT🡪  8 months:  22 children (44%) achieved  desensitization (1 g)  18 months:  36 children (72%) achieved desensitization (1 g) | | NA | |
| 1. **Peanut OIT studies** | | | | | | | | | | | | | | | |  |
| **Study** | | **Allergen** | **Study Design** | | **Number of participants** | **Patients’ age, median/mean**  **(range)** | | **Maintenance dose** | | **Duration of maintenance** | | **Results (Desensitization / Intention to treat)** | | **Sustained unresponsiveness (SU)** | |  |
| Hofmann et al, 2009 (90) | | Peanut | Open-label | | AG: 28 | 4.8 years (1.1-9.4 years) | | 300 mg PP | | 4-24 mo | | 20 children (71.4%) completed maintenance phase. | | NA | |  |
| Jones et al, 2009 (91) | | Peanut | Open-label | | AG: 39 | 57.5 months  (12-111 months) | | 300 mg PP  If peanut IgE >2 kU/L after 12 months on maintenance, dose was increased to 1800 mg PP | | 36 mo | | 27 children (69.2%) passed 3.9 g PP- OFC  10 children withdrew | | NA | |  |
| Blumchen et al, 2010 (92) | | Peanut | Open-label | | AG: 23 | 5.6 years  (3-14 years) | | 500 mg peanut | | 2 mo | | 14 children (61%) reached maintenance dose.  1 child could tolerate 0.2 mg peanut during maintenance.  8 children withdrew | | Avoid time: 2 w  In all children (n=14), median threshold dose significantly increased (1 g peanut) from baseline (0.19 g peanut) | |  |
| Varshney et al, 2011 (93) | | Peanut | Randomized, placebo controlled | | AG: 19  CG: 9 (placebo) | 7 years  (3-11 years) | | 4000 mg PP | | 48 w | | AG: 16 children (84.2%) passed OFC. (5000 mg PP)  3 children withdrew  CG: Median cumulative dose ingested by 9 children was 280 mg (range: 0-1900 mg) PP | | NA | |  |
| Anagnostou et al, 2011 (94) | | Peanut | Open label | | AG: 22 | 11.0 years (4-18 years) | | 800 mg PP | | 30 w | | 19 children (90.5%) reached maintenance dose.  6 weeks: 12 children (54.5%) passed OFC (2.6 g PP).  30 weeks: 14 children (63.6%) children passed OFC (6.6 g PP). | | NA | |  |
| Schneider et al, 2013 (95) | | Peanut | Open label  (Omalizumab was administered as an adjuvant) | | AG: 13 | 10 years  (7-15 years) | | 4000 mg peanut flour | | 12w | | 12 children (92.3%) passed OFC (8000 mg peanut flour). | | NA | |  |
| Vickery et al, 2014 (96) | | Peanut | Open-label | | AG: 39 | 1-16 years | | 4000 mg PP | | 5 years | | 24 children completed the protocol and evaluated for SU. | | Avoid time: 4 w  12 children (50%) passed OFC. (5000 mg PP) | |  |
| Anagnostou et al, 2014 (97)  (STOP II study) | | Peanut | Randomized, controlled, cross-over | | AG: 49  CG: 50 (avoidance) | 12.4 years  (7-16 years) | | 800 mg PP | | 6 mo | | AG: 33/39 children reached maintenance phase.  24 of 39 children (61.5%) passed OFC (1400mg PP).  10 children excluded from primary analysis.  CG: None of the children (0/46) passed OFC (1400 mg PP) at avoidance phase.  4 children withdrew  In crossover phase 41 of 45 children (91%) reached maintenance phase and 24/45 children (54%) passed OFC (1400 mg PP). | | NA | |  |
| Tang et al, 2015 (98) | | Peanut | Randomized, placebo-controlled  (Lactobacillus rhamnosus was administered as an adjuvant) | | AG: 31  CG: 31 (placebo) | 6.1 years  (1-19 years) | | 2000 mg PP | | 18 mo | | AG: 26 children (83.9%) passed OFC (4000 mg PP)  2 children withdrew  CG: 2 children (6.5%) passed OFC (4000 mg PP)  3 children withdrew | | Avoid time: 2-5 w  AG: 23 children (74.2%) passed OFC (4000 mg PP).  CG: 1 child (3.2%) passed OFC (4000 mg PP). | |  |
| MacGinnitie et al, 2016 (99) | | Peanut | Randomized, placebo controlled  (Omalizumab was administered as an adjuvant) | | AG: 29  CG: 8 | 10 years  (6-19 years) | | 2000 mg PP | | 12 w | | AG: 22 children (75.9%) passed OFC (4000mg PP).  CG: 1 child (12.5%) passed OFC (4000mg PP). | | NA | |  |
| Vickery et al, 2017 (100) | | Peanut | Randomized, controlled | | AG:  low dose: 20  high dose: 17  CG: 154  (historical cohort) | 28.5 months  (9-36 months) | | Low dose: 300 mg PP  High dose: 3000 mg PP | | 36 mo | | Low dose: 17 children (85%) achieved desensitization.  High dose: 13 children (76%) achieved desensitization. | | Avoid time: 4 w  Low dose: 17 children (85%) passed OFC (5000 mg PP).  High dose: 12 children (71%) passed OFC (5000 mg PP). | |  |
| Kukkonen et al, 2017 (101) | | Peanut | Controlled | | AG: 39  CG: 21 (placebo) | 8.3 years  (6-18 years) | | 800 mg PP | | 1 mo | | AG: 33 children (85%) reached maintenance dose.  26 children (67%) passed OFC (1255 mg PP)  CG: None of the children passed OFC (1255 mg PP) | | NA | |  |
| Hsiao et al, 2017 (102)  (Follow up study of Tang et al, 2015 (98)) | | Peanut | Randomized, placebo controlled  (Lactobacillus rhamnosus was administered as an adjuvant) | | AG: 24  CG: 24 (placebo) | 12.1 years (SD: 2.4 years) | | NA | | 4.2 years follow-up after treatment | | AG: 16 children (67%) continued eating peanut.  CG: 1 child (4%) continued eating peanut. | | Avoid time: 8 w  AG: 7 of 12 children (58%) passed OFC (4000 mg PP).  CG: 1 of 15 children (7%) passed OFC (4000 mg PP) | |  |
| Bird et al, 2018 (103)  (PALISADE study) | | Peanut | Randomized, placebo controlled (phase 2) | | AG: 29  CG: 26 (placebo) | 7 years  (4-26 years) | | 300 mg characterized PP (AR101) | | 2 w | | AG: 23 children (79%) passed 443 mg PP OFC.  18 children (62%) passed 1043 mg PP OFC.  CG: 5 children (19%) passed 443 mg PP OFC.  None of the children passed 1043 mg PP OFC. | | NA | |  |
| Nagakura et al, 2018 (104) | | Peanut | Open label | | AG: 22  CG: 11 (historical) | 8.5 years  (5-18 years) | | 795 mg PP | | >3 mo  Total duration: 2 years | | AG: All children achieved desensitization. | | Avoid time: 2 w  AG: 15 children (68%) passed OFC (795 mg PP).  CG: 2 children (18.1%) passed OFC (795 mg PP). | |  |
| Vickery et al, 2018 (105)  (PALISADE study) | | Peanut | Randomized, placebo-controlled | | AG: 416 (372 were 4-17 years)  CG: 139 (124 were 4-17 years) (placebo) | 11.3 years  (4-55 years) | | 300 mg characterized PP (AR101) | | 12 mo | | AG: 250 of 372 children (67%) passed OFC (600 mg PP)  CG: 5 of 124 children (4%) passed OFC (600 mg PP) | | NA | |  |
| Blumchen et al, 2019 (106) | | Peanut | Randomized, placebo-controlled | | AG: 31  CG: 31 (placebo) | 6.6 years  (3-17 years) | | 125mg PP (range: 50-250 mg PP) | | 2 mo | | AG: 23 children (74%) passed 300 mg PP-OFC.  13 children (42%) passed 4500 mg PP-OFC.  CG: 5 children (16%) passed 300 mg PP-OFC.  1 child (3.2%) passed 4500 mg PP-OFC. | | NA | |  |
| Chinthrajah et al, 2019 (107)  (POISED study) | | Peanut | Randomized, placebo controlled | | AG:  Peanut-0 group: 60  Peanut-300 group: 35  CG: 25 (placebo) | 10 years  (7- 55 years) | | Peanut-0 group: 4000 mg PP for 104 w  Peanut-3000 group: 4000 mg PP for 104 w followed by either a lower dose of 300 mg PP. | | 156 w | | At 104 and 117 w:  Peanut-0 group: 21 children (35%) passed OFC. (4000 mg PP)  CG: 1 child (4%) passed OFC. (4000 mg PP) | | Avoid time: 52 weeks for peanut 0 group.  Peanut 0 group: 8 children (13%) passed OFC. | |  |
| Hourihane et al, 2020  (108)  (ARTEMİS study) | | Peanut | Randomized, placebo-controlled,  phase 3 trial | | AG: 132  CG: 43 (placebo) | 9.1 years  (4-17 years) | | 300 mg characterized PP (AR101) | | 3 mo | | AG: 77 children (58%) tolerated 1000 mg PP at exit OFC.  CG: 1 child (2%) tolerated 1000 mg PP at exit OFC. | | NA | |  |
| Vickery et al, 2021 (109)  (PALISADE study) | | Peanut | Open-label follow-on study | | AG: 256 (OIT continuing group)  Naïve Group: 102 (OIT started) | 4-17 years | | AG:  Cohort 1 (C1): 300 mg/daily  Cohort 2 (C2):  300 mg every other day for 4 w and 300 mg twice weekly for 24 w  Cohort 3:  3A: 300 mg/daily  3B: 300 mg/daily for 4w, 300 mg every other day for 24 w, 300 mg BIW for 24 w  3C: 300 mg/daily for 4w, 300 mg every other day for 24 w, 300 mg twice weekly for 24-56w | | C1, C2: 28 w  C3: 56 w | | Rates of negative OFC at the end of the study (2000 mg PP):  NG: 51.4%  C1: 48.5%  C2: 36.8 %  C3A: 80.8%  C3B: 45.5%  C3C: 42.9% | | NA | |  |
| **D. Other foods OIT studies** | | | | | | | | | | | | | | | |  |
| Elizur et al, 2019 (111) | | Walnut | Prospective cohort, controlled, cross-over | | AG: 55  CG: 18 (avoidance) | 7.9 years  (4-17 years) | | 1200 mg walnut protein | | 6 mo | | AG: 49 children (89%) achieved desensitization.  All children co-allergic to pecan were achieved desensitization to pecan.  CG: None of the children achieved desentization. | | NA | |  |
| Moraly et al, 2020 (112) | | Hazelnut | Retrospective study | | AG: 100 children | 5 years  (3-9 years) | | 416 mg hazelnut protein  (2 whole hazelnuts)/ 3 times a week | | Total duration of the study: 6-7 mo | | 34% of the children achieved desensitization (passed 1635 mg hazelnut protein-OFC) | | NA | |  |
| Nachshon et al, 2019 (113) | | Sesame | Real-world study | | AG: 60  CG:15 (avoidance) | 7.5 years  (4-17 years) | | 1200 mg sesame protein  (5 g tahini) | | 6 mo | | AG: 43 children (71.6%) passed OFC. (4000 mg sesame protein)  CG: None of the children passed OFC. (4000 mg sesame protein) | | NA | |  |
| Nowak-Wegrzyn et al, 2019 (114) | | Wheat | Randomized, placebo-controlled | | AG:20  CG: 23 (placebo) | 8.7 years  (4.2-22.3 years) | | Low dose: 1445 mg wheat protein  High dose cross-over phase: 2748 mg wheat protein | | 1 year | | AG:  1^st^ year: 12 children (52.2%) achieved desensitization. (4443 mg wheat protein)  2^nd^ year: 7 children (30.4%) achieved desensitization. (7443 mg wheat protein)  CG: None of the children desensitized.  CG crossed-over high dose OIT. After high dose OIT 21 children (57.1%) achieved desensitization. (7443 mg wheat protein) | | Avoid time: 8-10 w  13% of the children had SU. | |  |

AG: active group, CG: control group, DBPCFC: double-blinded placebo-controlled food challenge; EP: Egg protein; OFC: Oral food challenge; OIT: Oral immunotherapy; PP: Peanut protein;; SU: Sustained unresponsiveness
